# The SOS-framework (Systems of Sedentary behaviours): an international transdisciplinary consensus framework for the study of determinants, research priorities and policy on sedentary behaviour across the life course: a DEDIPAC-study

**DOI:** 10.1186/s12966-016-0409-3

**Published:** 2016-07-15

**Authors:** Sebastien F. M. Chastin, Marieke De Craemer, Nanna Lien, Claire Bernaards, Christoph Buck, Jean-Michel Oppert, Julie-Anne Nazare, Jeroen Lakerveld, Grainne O’Donoghue, Michelle Holdsworth, Neville Owen, Johannes Brug, Greet Cardon, on behalf of the DEDIPAC consortium, expert working group and consensus panel

**Affiliations:** Institute for Applied Health Research, School of Health and Life Science, Glasgow Caledonian University, Glasgow, G4 0BA UK; Department of Movement and Sports Sciences, Ghent University, Ghent, Belgium; Department of Nutrition, University of Oslo, Oslo, Norway; TNO, Leiden, The Netherlands; Leibniz Institute for Prevention Research and Epidemiology- BIPS, Bremen, Germany; Department of nutrition, University Pierre et Marie Curie, Institute of cardiometabolism and nutrition (ICAN), Pitie-Salpêtrière hospital (AP-HP), Paris, France; CARMEN, Inserm U1060, Université de Lyon 1, Inra U1235, Lyon, France; Department of Epidemiology and Biostatistics and the EMGO Institute for Health & Care Research, VU Medical Centre, Amsterdam, The Netherlands; Centre for Preventive Medicine, School of Health & Human Performance, Dublin City University, Dublin, Ireland; Public Health Section, School of Health and Related Research-ScHARR, The University of Sheffield, Sheffield, UK; Baker IDI, Melbourne, VIC Australia

**Keywords:** Sitting, Sedentary behaviour, Determinants, Youth, Adults, Older adults, Ageing, Life-course, System-based approach, Environment, Concept mapping, Policy, Europe, Public health

## Abstract

**Background:**

Ecological models are currently the most used approaches to classify and conceptualise determinants of sedentary behaviour, but these approaches are limited in their ability to capture the complexity of and interplay between determinants. The aim of the project described here was to develop a transdisciplinary dynamic framework, grounded in a system-based approach, for research on determinants of sedentary behaviour across the life span and intervention and policy planning and evaluation.

**Methods:**

A comprehensive concept mapping approach was used to develop the Systems Of Sedentary behaviours (SOS) framework, involving four main phases: (1) preparation, (2) generation of statements, (3) structuring (sorting and ranking), and (4) analysis and interpretation. The first two phases were undertaken between December 2013 and February 2015 by the DEDIPAC KH team (DEterminants of DIet and Physical Activity Knowledge Hub). The last two phases were completed during a two-day consensus meeting in June 2015.

**Results:**

During the first phase, 550 factors regarding sedentary behaviour were listed across three age groups (i.e., youths, adults and older adults), which were reduced to a final list of 190 life course factors in phase 2 used during the consensus meeting. In total, 69 international delegates, seven invited experts and one concept mapping consultant attended the consensus meeting. The final framework obtained during that meeting consisted of six clusters of determinants: Physical Health and Wellbeing (71 % consensus), Social and Cultural Context (59 % consensus), Built and Natural Environment (65 % consensus), Psychology and Behaviour (80 % consensus), Politics and Economics (78 % consensus), and Institutional and Home Settings (78 % consensus). Conducting studies on Institutional Settings was ranked as the first research priority. The view that this framework captures a system-based map of determinants of sedentary behaviour was expressed by 89 % of the participants.

**Conclusion:**

Through an international transdisciplinary consensus process, the SOS framework was developed for the determinants of sedentary behaviour through the life course. Investigating the influence of Institutional and Home Settings was deemed to be the most important area of research to focus on at present and potentially the most modifiable. The SOS framework can be used as an important tool to prioritise future research and to develop policies to reduce sedentary time.

**Electronic supplementary material:**

The online version of this article (doi:10.1186/s12966-016-0409-3) contains supplementary material, which is available to authorized users.

## Background

The Sedentary Behaviour Research Network defines sedentary behaviour (SB) as “any waking activity characterized by an energy expenditure ≤ 1.5 metabolic equivalents while being in a sitting or reclining posture” [1]. In modern society, adults and children increasingly spend extended periods of time sedentary at home, at work, in education, and during transport and leisure [2]. Recent evidence shows that extended periods of sitting have a negative impact on health and wellbeing, and are associated with risk of developing chronic diseases such as type 2 diabetes, cardiovascular diseases, osteoporosis, breast and colon cancer, and with premature death [3–10]. The problem of spending too much time in SB has been documented in children of all ages [11–13], adults [14] and older adults [15–17], which clearly shows the need of tackling this emerging public health problem across the life span. The European Joint Programme Initiative for action on diet, physical activity and health (DEDIPAC) [18] aims to address the global growing trend in physical inactivity [19] and increased sedentary time [2] and their associated social and economic cost. The objective of DEDIPAC is to create a unified transdisciplinary vision among stakeholders to foster meaningful breakthroughs in the understanding of the determinants of SB necessary to the development of programs, public health campaigns and policies to reduce SB [20].

Time spent sedentary is influenced and conditioned by multiple inter-dependent factors acting on multiple levels. To date, few and a very narrow range of factors focussing mostly on individual factors have been thought of or identified and even fewer investigated [21–24]. One of the challenges is to develop a common model and framework to guide future transdisciplinary research in the identification of key modifiable factors or cluster of factors and their interactions. This is essential to enable stakeholders and policy makers to plan and develop effective and sustainable solutions to reduce SB through the life course.

Currently, a single conceptual model has been proposed to facilitate the exploration of determinants of SB [20]. It is based on the social ecological framework [25–27] which theorises behaviour as result of the interplay between a person and his or her environment formed of nested spheres of influences. Ecological models commonly consider; individual (e.g., biological, psychological, behavioural aspects), interpersonal (e.g., family, friends, social networks), physical environment (e.g. access to facilities), and public policy factors (e.g., national, local laws and organisational rules) spheres [25–27]. The ecological model of SB provide a useful overview and enable to class determinants in different level of influence but has limitation for transdisciplinary research inherent to all ecological models [27]. First the ecological model of SB was developed on a theoretical basis from a single ontological view point rather than by using a formal methodology to engage multi-disciplinary views. Consequently, it does not provide a shared model emerging from transdisciplinary eminence and evidence. Second, while ecological models were a real breakthrough in acknowledging the complexity of the determinants of health behaviour, they rest on the epistemological assumption of hierarchical dependencies between spheres of influence. This limits their ability to fully capture the complexity of specific behaviours or understand the complex interplay between determinants [25–27]. Therefore the relationship between different determinants and in particular those at the more proximal and distal levels is not mapped. Finally, their applications to public health research in the last 20 years have focussed the attention of intervention and epidemiological research primarily on individual characteristics, with mitigated results because, conceptually, they place the individual at the centre [27]. Three systematic reviews conducted by the DEDIPAC KH indeed clearly show that the vast majority of research has focused on individual factors and has mostly neglected distal factors [21–24]. Consequently there is a need to develop a more agnostic framework based on transdisciplinary views, different conceptual approach and a formal methodology.

A system-based approach has been advocated as useful alternative, to overcome the limitations of an ecological models [23, 28, 29]. A system-based approach focusses on the interrelationship of parts (i.e., subsystems) and their dynamic functioning as a whole (i.e., system), rather than the individual. It incorporates the relationship between distal and proximal factors at different scales from micro to macro in the context of determinants and has received growing interest as a new paradigm for public health, with notable examples such as the Foresight model of obesity [30].

Therefore, the aim of this project was to fulfil the objective of DEDIPAC KH (DEterminants of DIet and Physical Activity Knowledge Hub) [18] of creating a shared transdisciplinary system-based framework of the determinants of SB, using a formal methodology merging transdisciplinary evidence and eminence.

The purpose of this framework is to 1) foster transdisciplinary system thinking,2) facilitate the identification of factors and cluster of factors influencing SB and 3) guide secondary analyses of existing data, 4) prioritise research and guide targeted interventions and policy.

## Methods

### Design

**Fig. 1:**
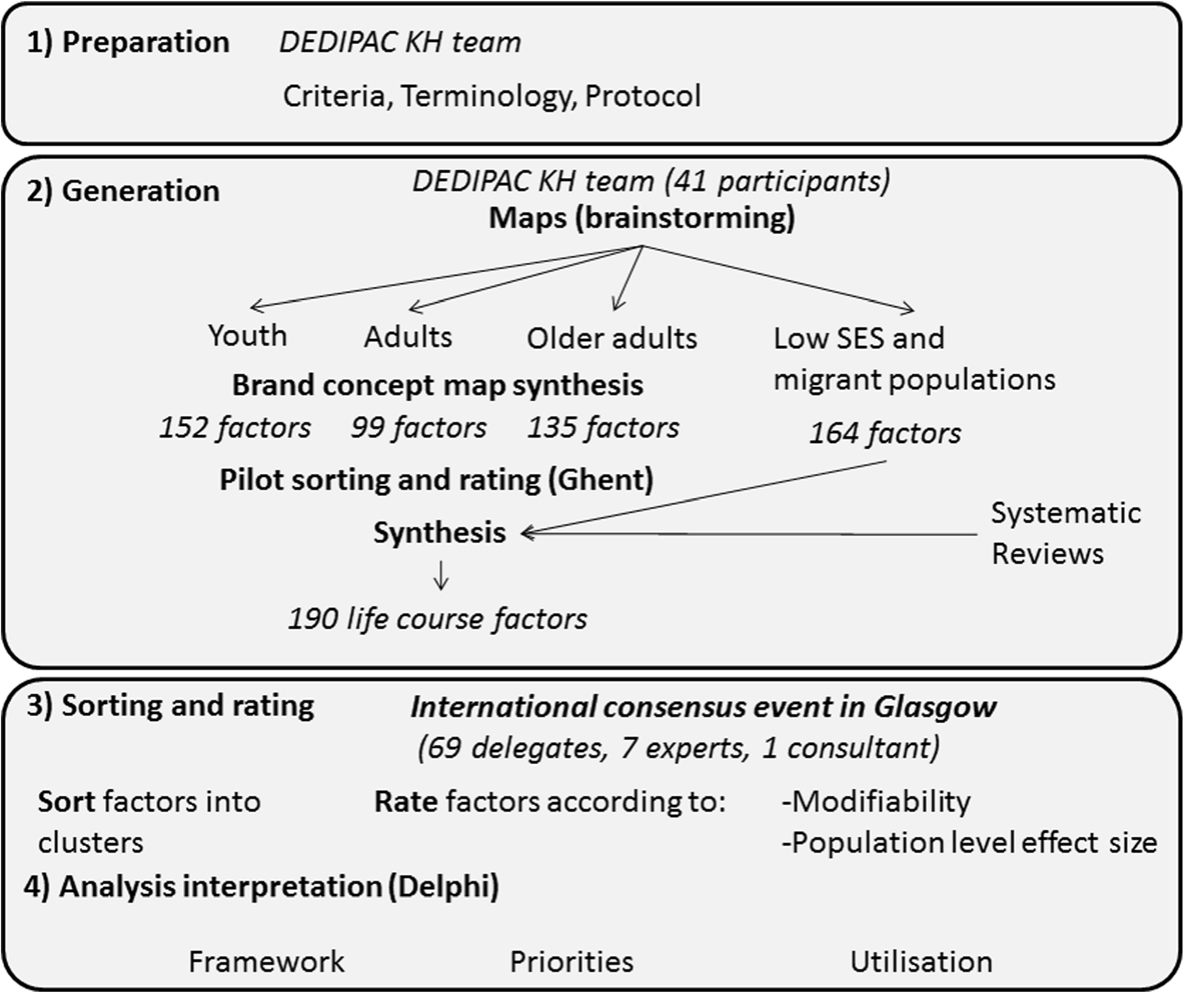
The concept mapping process

We used a structured consensus protocol based on concept mapping [31]. Concept mapping is a standardised mixed method, which combines qualitative opinions with multivariate statistical analysis to enable a group to gather and organise ideas into a conceptual framework. Concept mapping was originally designed for program evaluation and planning [32] but has also proven to be an effective method for synthesising expert opinions. It is particularly suited for defining and conceptualising complex public health systems with many interacting parts acting at different scales [28, 29, 33, 34]. Concept mapping involves four main phases (Fig. 1): (1) preparation, (2) generation of statements, (3) structuring (sorting and ranking), (4) analysis and interpretation. Details of the implementation of each of these phases are given below. The preparation and generation of statements were undertaken by the DEDIPAC KH team on Determinants of SB between December 2013 and February 2015. The structuring, analysis and interpretation phases were achieved during a two day consensus meeting in June 2015.

### Framework objectives and criteria

During the preparation phase, the following aims were set for the framework.

- Capture broad scientific **transdisciplinary** thinking.
- Gather an **exhaustive** list of all known factors and all potential factors (new factors and those for which evidence might be indecisive or not investigated at present).
- **Organise** these factors into a system that captures their thought relationship.
- Highlight areas of **priority and modifiability** within this system.

To achieve these aims, the protocol was set to adhere to the criteria below.

- The framework must emerge from a broad transdisciplinary scientific consensus.
- The framework must be as agnostic as possible.
- The framework must be as exhaustive as possible.
- The framework must be based on a structured process and sound methodology.

#### Preparation

During the preparation phase, the aims of the framework were defined by the DEDIPAC KH team in line with a set of criteria that the framework had to fulfil. Relevant literature about concept mapping [31, 32] and system-based approaches [33, 35] was shared amongst the team beforehand to increase capacity and a common understanding. A common terminology and common definitions of important terms were developed and distributed to facilitate multidisciplinary communications. In particular, the concept mapping jargon was modified to align with the aim of the project. Concept mapping is based on a set of “statements”, since the aim of the project was to define a system-based model of determinants we replaced the term “statement” by the term “factor” as the most agnostic word to qualify an entity associated with SB (e.g., determinant, correlate, moderator, mediator). The Sedentary Behaviour International Taxonomy was adopted to provide common definitions of SB [36].

Finally, a protocol was established to structure and standardise the whole process and a guide detailing how to contribute was written for the participants [37].

#### Generation of the list of factors

The list of factors was established through an iterative process combining eminence and evidence through the use of three sources of information: expert opinion of the DEDIPAC KH team on determinants of SB, three systematic reviews of determinants of SB produced by the same team [21–24] and expert opinion of the DEDIPAC KH team working on social inequality and ethnic minority populations. The latter input ensured that the emerging framework also accounted for social and ethnic diversity in populations and factors specific to ethnic minorities, vulnerable groups and socially disadvantaged strata of society.

The DEDIPAC KH team on the determinants of SB was asked to individually establish exhaustive lists of factors that they thought could influence SB. Participants were encouraged to go beyond the current evidence base and capture all potential factors they could think about whether they had been investigated or not. To facilitate this process, individuals developed lists using categories based on the ecological model of SB [38] separately for youths (below the age of 18), adults and older adults (people aged 65 and over). Individuals then worked in teams within their institutions to produce “free hand” diagrams of how these factors might be interconnected using graphical software (PREZI).

**Fig. 2:**
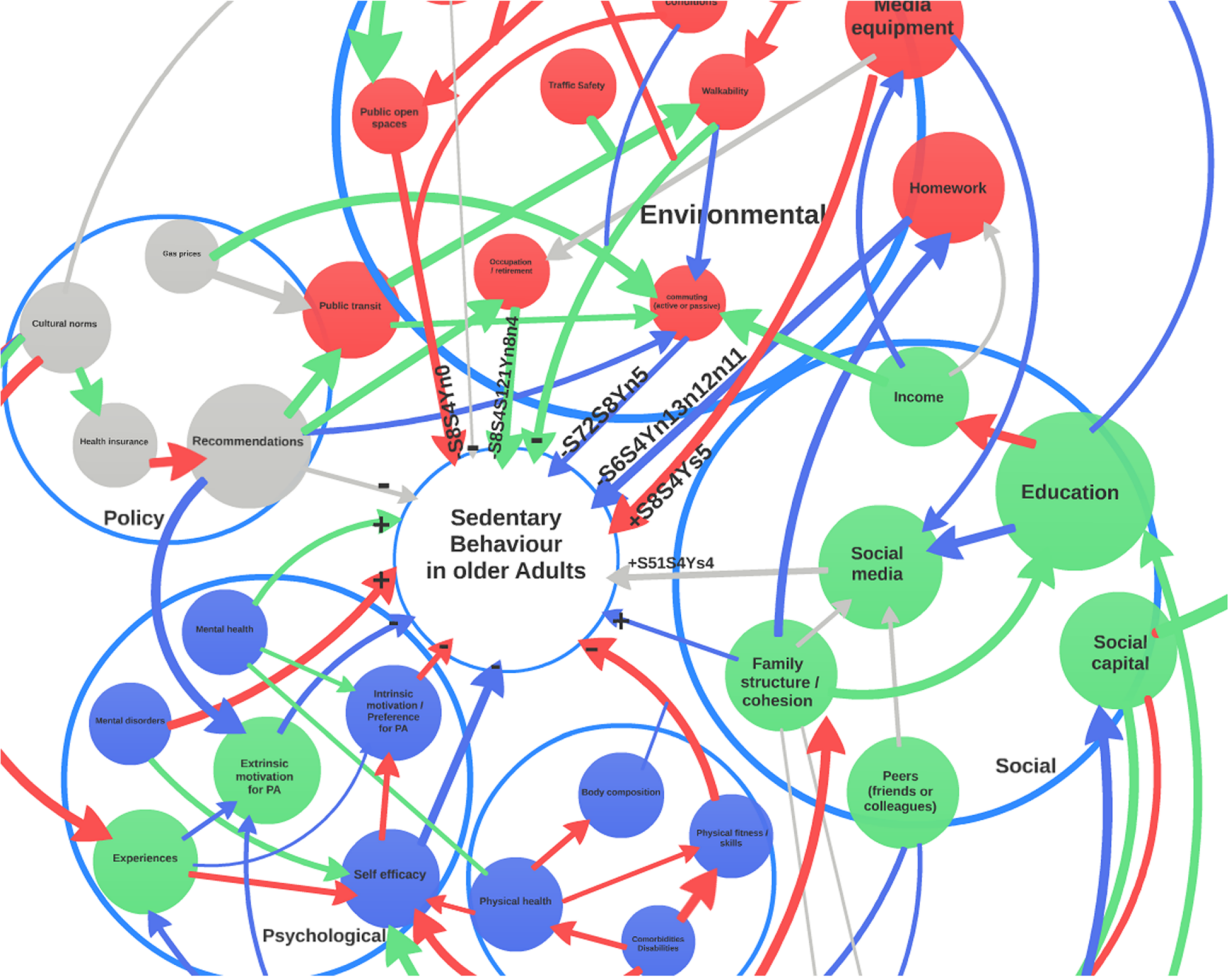
Example for illustration of free hand system map drawn by experts

In these diagrams, participants were asked to code their views of the direction, empirical and theoretical strength of the relationship through the colour and thickness of arrows linking factors. This was to encourage participants to start structuring factors into a system thinking approach, harvest a wide variety of potential factors and map relationships between factors. These diagrams were analysed and synthesised using a brand concept mapping technique [39] into one diagram (Fig. 2) and one list of factors was generated for each age group, namely for youth, adult and older adult populations.

In September 2014, the DEDIPAC KH team on determinants of SB met for a workshop and undertook a concept mapping exercise aimed at reducing the size of the list of factors, evaluating concept mapping software and piloting the procedure of the main consensus event. During the meeting, the team sorted factors into related clusters and rated the factors’ modifiability, theoretical effect size and priority for research, a five point Likert scale. The statistical analysis and interpretation of the resulting concept maps produced three lists of ranked factors (one for each age group), which were then merged into a single life-course list. This list was then enriched with factors identified to be especially relevant to social inequality and ethnic minority populations by the DEDIPAC KH team working on this. Finally, the findings from the three systematic reviews [21–23] complemented the list. Duplicates were then removed. A three round Delphi process amongst the DEDIPAC KH team on determinants of SB yielded the final list of factors, each with a precise wording and an accompanying definition, both of which were used in the main consensus concept mapping event.

### Consensus event

The consensus event was held at Glasgow Caledonian University, Scotland, on June 8^th^ and 9^th^ 2015, as a satellite meeting to the International Society of Behavioural Nutrition and Physical Activity annual conference. Open invitations to take part were issued through relevant networks and scientific societies (e.g. DEDIPAC, Sedentary Behaviour Research Network, International Society of Behavioural Nutrition and Physical Activity, Health Enhancing Physical Activity, International Society for the Measurement of Physical Behaviour, European Association for the Study of Obesity), as well as direct contact to known experts.

This meeting was facilitated by the DEDIPAC KH team on determinants of SB, an expert working group and a concept mapping consultancy (Minds21). Concept mapping expertise was provided by Minds to One a consultancy company which developed and online concept mapping tool called Ariadne (www.minds21.org). An Expert Working Group was recruited amongst to represent expertise in system-based approach, sedentary behaviour in different age groups and settings. The responsibility of the Expert Working Group was to offer eminence, provide a critical overview, facilitate debates and assist the DEDIPAC KH in summarising the concept mapping exercise. Before the meeting, all participants received a document explaining its aim and procedure, a reading list of relevant literature on system-based approach and concept mapping, common terminology and definitions, and the list of factors with their definitions. The event was video recorded to enable analysis of any inconsistencies. The meeting opened with members of the Expert Working Group giving their opinion about a system-based approach and determinants of SB. The DEDIPAC KH presented the results of the three systematic reviews of determinants of SB in youths, adults, and older adults [21–24]. In addition, the findings of a mapping review of determinants specific to social inequality and ethnic minority populations were presented. The procedure, common terminology and list of factors were explained and discussed in an open question and answer plenary session before the sorting and ranking of the factors began.

### Participants

Three distinct groups of participants took part in the consensus process: 1) members of the DEDIPAC KH team on determinants of SB undertook the preparation and generation phase and took part in the whole process, 2) an expert scientist working group was recruited directly based on publication records in the field of SB research, their respective field of expertise and focus on specific stage of the life course, and 3) scientist who self-selected to attend the two day consensus event.

#### Sorting and ranking

Sorting and ranking occurred during the first day of the event using the concept mapping software Ariadne (Minds21). In order to have a validated consensus framework, it was decided to let participants sort and rate a random sample of factors. This was discussed with Prof. Trochim at a lecture on the 26th of March 2015 in Utrecht (the Netherlands). The concept mapping consultant from Minds to One was also present at this discussion, and a decision was made to have a random sample of 70 factors. The decision to have a random sample of 70 factors was based on the fact that we would then have the strongest framework within the short timeframe (one day to explain the process and perform the sorting and rating) and with the number of people attending the consensus meeting. The number 70 was arbitrary and could have been a few more or less. Each participant was given a login to the software and asked to make clusters of a random sub-sample of 70 factors from 190 in the list and rate the factors on a 5 point Likert scale according to four criteria: modifiability and the strength the effect of the factor, combining effect size and prevalence, for youths, adults and older adults. They were also asked to name the clusters they created during sorting.

#### Data analysis and concept map

At the end of day 1, the DEDIPAC KH team, the expert working group and the concept mapping consultant (*n* = 12) analysed the data and prepared the utilisation phase. Multidimensional scaling statistics were used in Ariadne [31] by the DEDIPAC KH and Expert Working Group to generate a concept map of distinct clusters that reflected all delegates’ suggestions. The validity of the emerging clusters was tested via the Stress Test [40]. A list of four possible system names was generated for each cluster based on participants’ suggestions. Ratings were analysed to create a list of factors ranked by priority for research (based on equally weighted modifiability and life-course population level effect ratings) for each cluster. Average scores from these lists were computed for the four criteria for each cluster. This information was used to generate slides for presenting the results and to capture opinions about the utilisation and priority for research.

### Utilising the map to define the systems framework and priorities

On the second day, participants were presented with the results of the sorting and ranking step and through a Delphi process defined the framework and priority for research. Participants were asked specific questions based on the results and an open discussion followed. The key points of the discussion were summarised and fed back to the participants, until no more objections or further issues were raised with the results. At this point, participants were asked to vote via the Turning Point software and were given the results in real time. As a consequence, the voting options were sometimes modified (e.g. wording of options for cluster names changed until the panel was satisfied it represented its views or the views of a majority of participants). Participants anonymously voted for the system name of each cluster, priority for research of each system and priority for research of factors within each system. Consensus level was pre-set at 70 % of the participants for dichotomous choices and as a majority vote for multiple choices. For sense checking participants were also if they agreed that the final framework captures a system-based map on determinants of SB, if they found it was useful and if they wanted to be involved in further development.

## Results

### Consensus panel characteristics

Table 1 below provides an overview of the characteristics of the participants in the concept mapping process at the different stages, in terms of numbers, field of expertise and nationality (place of work).

**Table 1:** Consensus panel characteristics at the different concept mapping stages

	Generation Brainstorming	Pilot	Consensus Event
Participant Numbers and Roles	32 (SB team) 9 (Inequalities team)	13 (SB team) 9 (Inequalities team)	69 delegates 7 invited experts 1 Concept mapping consultant
Fields of expertise	Ageing science Economics Epidemiology Exercise physiology Gerontology Health inequalities Health promotion Migration and public health Nutrition Physiotherapy Psychology Public health Social sciences Sociology Sport and exercise science Statistics	Ageing science Economics Epidemiology Exercise physiology Gerontology Health promotion Nutrition Physiotherapy Public health Sport and exercise science Statistics	Ageing science Anthropology Behavioural sciences Bioengineering Biostatistics Clinical biochemistry Clinical sciences Developmental psychology Economics Epidemiology Ergonomics Gerontology Health promotion Life sciences Measurement science Medicine Movement science Nutrition Paediatrics Physiotherapy Psychology Public health Rehabilitation sciences Social sciences Sport and exercise science Sports psychology Statistics Translational research
Countries	Belgium France Germany Ireland Italy Netherlands Norway UK	Belgium France Germany Ireland Italy Netherlands UK	Australia Belgium Brazil Finland France Germany Hong Kong Ireland Italy Netherlands Norway UK USA

### Factors list

In the preparation phase, a total of 550 factors were listed by the participants; 152 for youths, 99 for adults, 135 for older adults and 164 relevant to health inequalities in ethnic minority populations. Of these, 344 factors were either duplicates or very similar constructs and were therefore merged. For example, factors such as “Mobility Issues” and “Loss of Physical Function” were merged into a single factor “Mobility Issues: issues relating to the physical ability to move or be moved freely and easily”. Similarly factors such as “Design of Classroom”, defined for youths, “Work: Building Organisation”, defined for adults, and “Care Home Design”, defined for older adults were pulled across the life course as “Physical Organisation and Furniture of Place of Education/Work/Care: the physical and aesthetic design of place of education/work/care”. A further 16 factors were dropped because a precise definition could not be found or agreed upon (e.g. “Romantic Dyad”). The final list of 190 factors considered in the Concept Mapping exercise is given in the Additional file 1: Table S1 together with their definition.

### Concept map

**Fig. 3:**
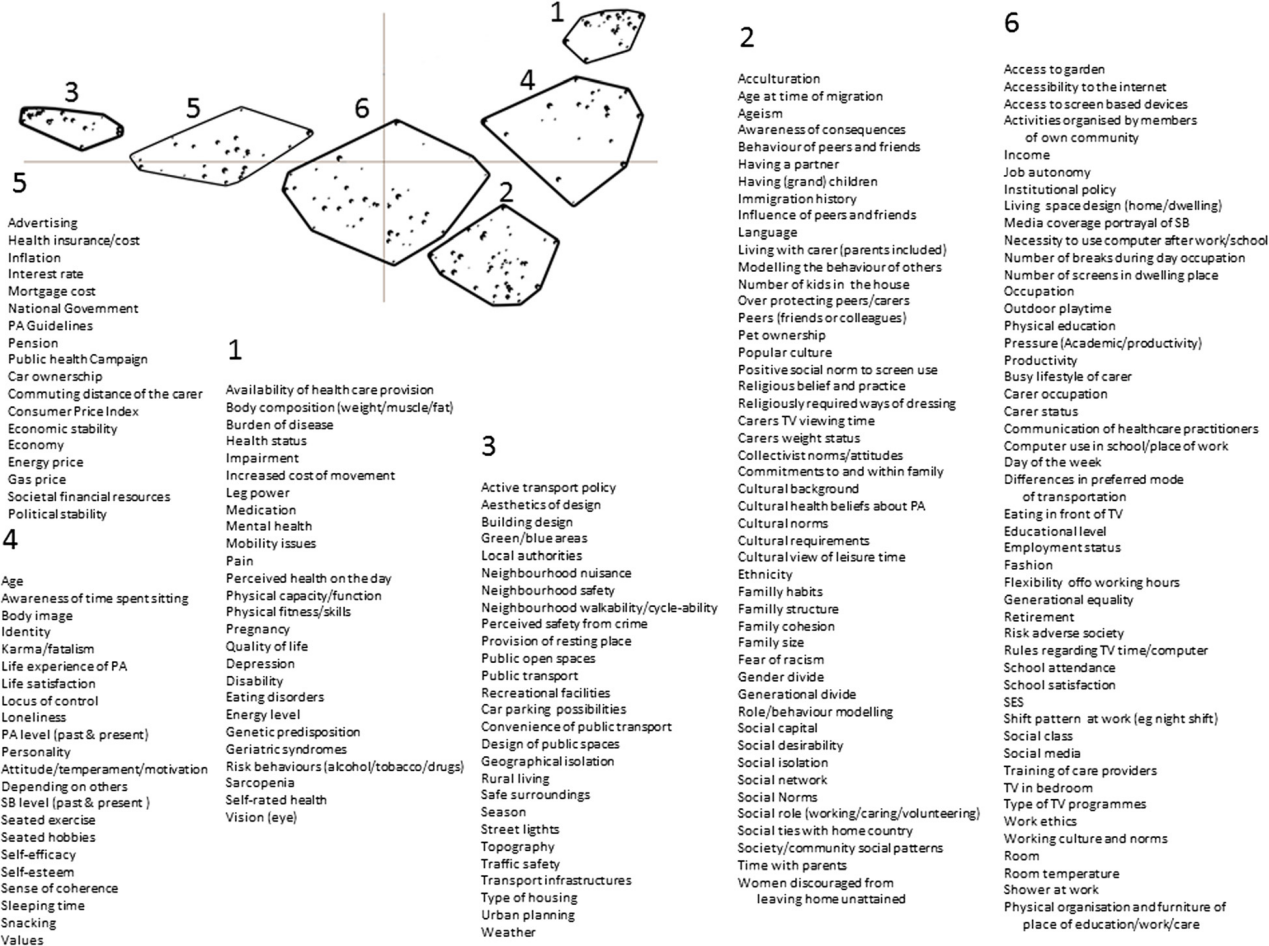
Concept map with position (dots) and list of factors within the six clusters

Analysis of the sorting performed during the consensus meeting revealed that a concept map with six cluster solution encapsulated the consensus. The map and factors included in each cluster are shown on Fig. 3. Based on the Stress Test, these six clusters captured 76 % of variance in opinion between experts.

### System-based framework

**Fig. 4:**
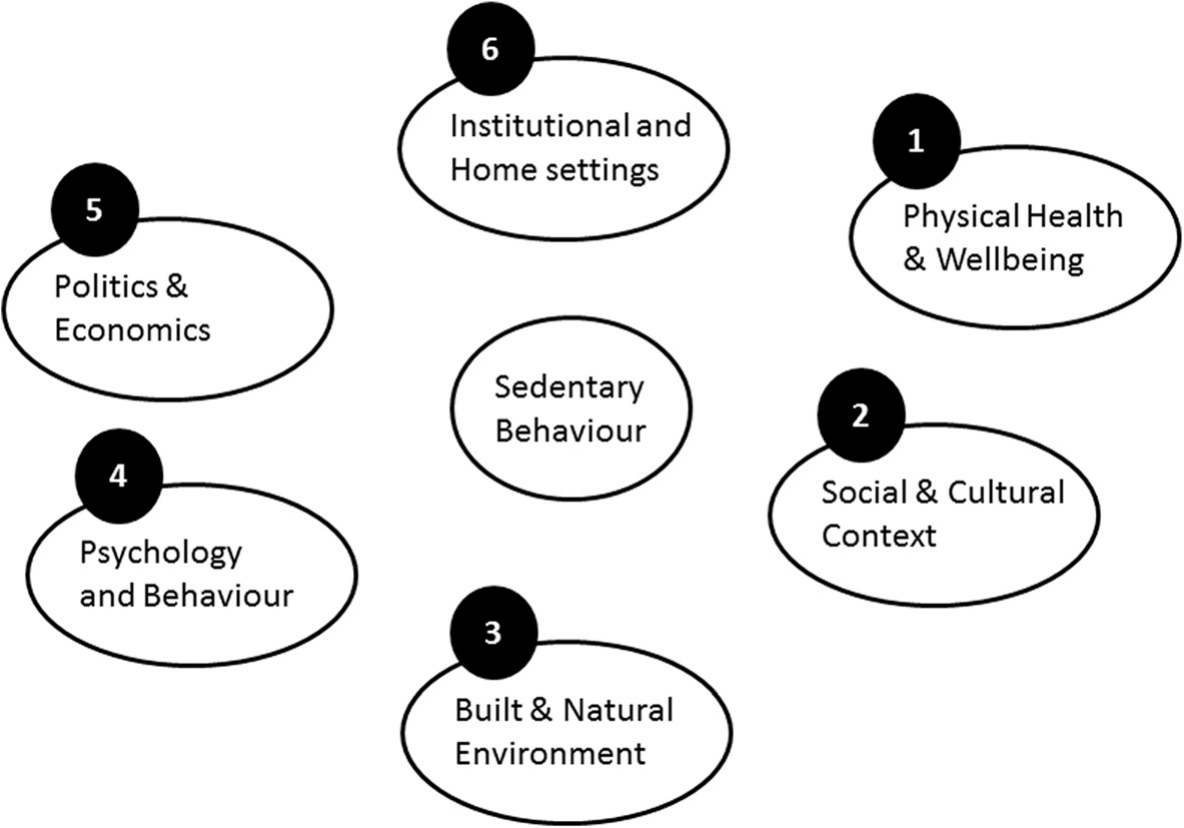
Framework

The final framework, shown in Fig. 4, was developed based on this map. Consensus was obtained about the name of each cluster; Physical Health and Well-Being (71 % consensus), Social and Cultural Context (59 %), Built and Natural Environment (65 %), Psychology and Behaviour (80 %), Politics and Economics (78 %), Institutional Settings (78 %). In the end, 89 % of the participants expressed the view that this framework captures a system-based map of determinants of SB. Furthermore, 89 % also expressed the view that this framework is useful and 90 % signified that they want to get involved in further developments.

### Cluster definitions

Through review and discussion of the included factors, the following definitions for each cluster were developed.

#### Physical Health and Wellbeing

Cluster encompassing everything related to an individual/group’s health and wellbeing, including (but not limited) to their personal health status. For example, this cluster also covers systems for provision of health care or health enhancing facilities.

#### Social and Cultural Context

Cluster referring to the social environment that individuals/groups live in, the culture they were educated in and interact with.

#### Built and Natural Environment

Cluster referring to the physical environment that individuals/groups live in and interact with. This includes the natural environment factors such as weather or built environment such as the physical layout of towns.

#### Psychology and Behaviour

Cluster referring to individual’s/group’s psychological and behavioural traits such as motivations and attitudes.

#### Politics and Economics

Cluster encompassing political and economic factors that influence the civic life of individuals/groups at international, national, regional and individual scales.

#### Institutional and Home Settings

Cluster encompassing all factors influencing the physical and human organisation of institutions (e.g. the home, schools, workplace, and care homes) individuals/groups live in or interact with.

### Research priorities

A 92 % consensus was obtained about research priorities between clusters with research on institutional settings ranking first (Table 2). Consensus was not achieved about research priorities between factors within a cluster. However analysis of the ranking enabled to identify potential promising factors to investigate. Additional file 1: Table S2-S4 show factors with the highest combined modifiability and population-level effect size scores for the three age groups; youths, adults and older adults across the life course.

**Table 2:** Research priority ranking per cluster

Cluster research priority rank	Cluster
4	Physical Health and Wellbeing
5	Social and Cultural Context
3	Built and Natural Environment
2	Psychology and Behaviour
6	Politics and Economics
1	Institutional and Home Settings

## Discussion

We established a shared system-based framework and research priorities for the study of determinants of sedentary behaviour across the life course. This the first framework and set of research priorities developed using a formal consensus methodology drawing upon a wide transdisciplinary evidence and eminence.

### The SOS framework

The concept mapping process enabled the development of the system-based SOS (Systems of Sedentary behaviours) framework for the determinants of SB. The SOS framework provides a shared transdisciplinary model of the complexity of the web of factors that influence SB, across the life course, as the interaction of six main clusters. The health and wellbeing of individuals/groups, their psychology and behaviour, the culture and social context that individuals/groups are immersed in, the built and natural environment they live in, the settings of the institutions that individual/groups interact with (e.g. the workplace, school, place of care, towns, home) and the politics and economics.

The SOS framework builds upon but addresses the conceptual limitations of ecological model proposed by Owen et al. [20]. First, it was developed using formal methodology drawing from the latest evidence and transdisciplinary eminence rather than theory emerging from a single discipline (behavioural epidemiology). Second, it removes focus on the individual and instead points directly at systems that influence behaviour of individuals, groups and populations. Third, it removes the implied assumption of hierarchy between determinants present in ecological models. Finally, the framework integrates and is valid across the life course. Overall the SOS framework provides a more pragmatic and transdisciplinary shared model compared to the ecological model.

### Implications of the framework

The SOS framework implies a paradigm shift in the way we view, conceptualise and study factors that determine or condition sedentary behaviour. Instead of reducing determinants of SB to discrete factors organised in conceptual levels and seeking how these vary at the individual level, the SOS framework focuses our attention on understanding how six clusters of factors interact synergistically to promote or prevent sedentary behaviour. Traditional reductionist approaches facilitated by ecological models are ill-equipped to deal with the complexity of the web of influence determining SB, some authors actually consider them counter-productive and inefficient [29, 41]. System-based thinking supported by the SOS framework offer a complementary avenue which might allow significant breakthrough. The framework “de-silo’s” research on SB and provides an agnostic (not strongly theoretically constrained or model-biased), shared view of what potentially determines SB, enabling further development of transdisciplinary research. All reviews clearly show that mostly individual factors have been identified. The framework conceptually shifts emphasis away from the individual and toward system and clusters of factors which will enable the identification of factors and their inter-relationships. By not considering the individual as central, the SOS framework also enables to plan investigation about what determines group behaviours.

Similarly, the complexity of the problem highlighted by the framework should encourage innovative approaches to data analysis. In particular, techniques should be sought that can deal rigorously with such complexity and tightly correlated sets of factors. In this respect, Bayesian Network and dynamical simulation approaches have been advocated over regression techniques [42].

### Utilisation of the framework

A recent review within DEDIPAC on determinants of SB highlighted that most studies tend to concentrate on easily measurable, non-modifiable, individual-level parameters [23]. The SOS framework provides an overview of the complexity of SB that can guide the development and promotion of transdisciplinary studies probing the determinants of SB, beyond this limited focus.

During the consensus process it emerged that the framework holds promise as a tool to support the development of policy and complex interventions to reduce SB via actions on its determinants. In this respect, the framework makes it easier to identify key gaps in knowledge and to engage stakeholders in the co-creation and development of interventions and policies through helping them to identify the most relevant set of factors through which they could play a role in influencing determinants of SB. For example, the framework was used as a basis to develop solutions to reduce sedentary behaviour in stroke survivors at the 2015 UK Stroke Forum. Clinicians, researchers, carers and NHS trust managers used the framework to identify factors they could address in practice within three different settings of acute, rehabilitation and community care. Finally, considering that SB is influenced by such a complex web of factors, interactions and confounders, seeking only to identify relationships between them might be an arduous and lengthy task. Alternatively, the framework could be used in a more solution orientated manner [41], thereby providing a quicker route to developing effective policies and interventions that account for the complexity of SB. It could be used to develop scenarios to identify levers for change and potential strategies to use them at different life stages and in different contexts such as the workplace, school or in care settings [43].

### Life course aspect of the framework

The framework was developed with the life course in mind and through a process which acknowledged the life course through three life stages. The framework is valid dynamic model through the life course. The system of six clusters that drives sedentary behaviour remains the same, however the interplay between clusters, the importance of each cluster and the factors within each cluster will change. For example the health cluster is likely to take more importance in later life. Similarly the institutional and home settings cluster is likely to shift from school to work institutions as people transit from childhood to adulthood.

### Future developments

The framework should be operationalised for specific stages of the life course and for particular contexts such as, workplaces, schools, and older people’s care homes. This reflects – in a more comprehensive manner – the ‘behaviour settings’ in the ecological model of SB [20]. The results of this consensus process can be used as a starting point to develop more specific system models as the Foresight map of obesity [30]. To achieve this, the process of development of the SOS framework should be reversed. New lists of factors for each cluster and sub-cluster could be defined in specific contexts and for specific population and applications. The list of factors we developed and their clustering should not be taken as a final definitive listing and categorisation, as their purpose was primarily to map, in the SOS framework, the breadth of factors driving SB. Future systematic reviews of the evidence base should be mapped against this framework.

### Research priorities

Priorities for research were identified based on modifiability and potential for change of SB determinants at the population scale. Investigating the influence of institutional settings was deemed to be the most important area of research to focus on at present. Some potential promising factors were identified at all three life stages (Additional file 1: Table S1-S3) based on the fact that they were deemed the most modifiable and could have largest effect size at the population scale. Care should be taken in interpreting these results as a firm consensus about research priority regarding factors themselves. However, the consensus process revealed that prioritising discrete factors it is of little value at this point in time. Identifying and tackling discrete factors or group of factors should be done by operationalising for specific population, life stages or context as described above.

Instead, emphasis should be put on engaging with the complex and pervasive nature of SB and “de-silo” research by prioritising transdisciplinary investigations and interventions. In particular, the scope of existing research should be widened. In particular, research on the influence of politics, economics and policy has the potential to make strong and much-needed contributions in this field.

### Were criteria and aims met?

The conduct of the consensus process fulfilled the criteria set by the DEDIPAC KH at the beginning of the project and this was validated by ultimate feedback from the participants when they were asked to express their opinion about the process. The framework that emerged from the consensus also met the majority of our aims. It captures and organises in a system an exhaustive list of potential factors to consider as determinants of SB, this from a very broad transdisciplinary perspective. The process highlighted areas of priority for transdisciplinary research. However, a detailed mapping of the relationship between the cluster and factors was unfeasible. During the consensus process, there was broad agreement among participants that interaction between clusters is implicit and does not need to be represented. In addition, it was also recognised that factors in each cluster change with the stages of the life course and that their interactions will also change depending on context and population. It was agreed that developing the framework into a general fully deterministic model is not possible at present and might actually be counterproductive. It was felt that the value of the map did not reside in it being more detailed but in the paradigm shift it entails and how it captures a framework that can be operationalised for different uses. In addition, experience from the Foresight model of obesity, showed a full system map proved less useful for public health practice than a simpler one [30, 43].

### Strength and Limitations

One of the strengths of this study is that it relied on a thorough and structured formal consensus process. The consistency and rigor of the concept mapping protocol that was developed and implemented, adhered to guidelines for best practice [40] and had input from a large multidisciplinary team within the DEDIPAC KH.

The main limitation of the study is that participants, except the DEDIPAC KH team on determinants of SB and expert panel, self-selected into the consensus process. Consequently, we did not have a completely balanced distribution of expertise, disciplines and countries involved. Nonetheless, we achieved a broad multidisciplinary involvement. Few studies manage to engage this level of plurality and multidisciplinary expertise, nor to gather input and generate outcomes from discussion between disciplines as far ranging as anthropology, economics and rehabilitation science. While not all countries in the world were represented, the majority of countries with a high prevalence of sedentary time were represented and some countries where this public health problem is emerging, such as Brazil, were also present. In addition, many internationally leading experts in SB attended the meeting and contributed to the development of the SOS framework.

Policy makers and practitioners were not present in the process. This was intentional. Here we developed a theoretical, generic and transdisciplinary framework across the life course based on scientific eminence and evidence. We wanted to keep it free of political and pragmatic agendas and issues that policy makers or practitioner might have brought to the table at this stage. In addition, sedentary behaviour is so ubiquitous that it touches on all aspect of daily civil life. Therefore to be successful a consensus process would have required including policy makers and practitioners from a very wide area of civic life, which would have been 1) very difficult to achieve 2) premature as we did not know which systems and aspect of civic life to focus on. As described in the future work section policy makers and practitioners should be consulted in the future utilisation of the framework.

## Conclusions

A system-based framework for identifying the determinants of SB through the life course was established through an international multidisciplinary consensus process. The SOS framework describes SB as the complex interaction of six primary clusters (or sub-systems) of determinants: Physical Health and Wellbeing, Psychology and Behaviour, Built and Natural Environment, Social and Cultural Context, Institutional Settings and Politics and Economics. The framework can act as an important tool to prioritise future research and develop solutions to reduce sedentary time. Within the framework, understanding in more detail the role of Institutional Settings and their relationships with other clusters was identified as the most important priority in the short term. SB research should widen its scope and adopt a more transdisciplinary approach in order to engage with the complex web of influence determining SB.

## Abbreviations

BMI, Body Mass Index; SB, sedentary behaviour; SES, socio-economic status; SOS, systems of sedentary behaviour

## Additional file

Additional file 1: Table S1. List of factors. **Table S2**. Factors identified in each cluster as having combined highest modifiability and population level effect size in youths. **Table S3**. Factors identified in each cluster as having combined highest modifiability and population level effect size in adults. **Table S4**. Factors identified in each cluster as having combined highest modifiability and population level effect size in older adults. (DOCX 47 kb)
