# Supplementary material for: The SOS-framework (Systems of Sedentary behaviours): an international transdisciplinary consensus framework for the study of determinants, research priorities and policy on sedentary behaviour across the life course: a DEDIPAC-study
Source: Int J Behav Nutr Phys Act. 2016 Jul 15;13:83. doi: 10.1186/s12966-016-0409-3 (PMC4947275; doi:10.1186/s12966-016-0409-3)
Supplement: Additional file 1: Table S1. — List of factors. Table S2. Factors identified in each cluster as having combined highest modifiability and population level effect size in youths. Table S3. Factors identified in each cluster as having combined highest modifiability and population level effect size in adults. Table S4. Factors identified in each cluster as having combined highest modifiability and population level effect size in older adults. (DOCX 47 kb) [file 12966_2016_409_MOESM1_ESM.docx]

**Supplementary material**

**Table S1: Factors list with definition used in the consensus event**

| Nbr | Short Label | Definition |
| --- | --- | --- |
| 1 | Ability to use health care | Ability to use health care: Whether a person is able to access health care (e.g. able to pay for it if private or able to physically or culturally integrate with health care system) |
| 2 | Availability of health care provision | Availability of health care provision: Whether health care provision is available to a person (e.g. whether they have a health care system) |
| 3 | Access to garden | Access to garden: Whether a person has a garden, is able to access the garden |
| 4 | Access to screen based devices (computer, video games etc..) | Access to screen based devices: Whether a computer (video games) are available in the environment/house; whether a person is able to access these screen based devices without having to ask permission |
| 5 | Accessibility to the internet | Accessibility to the internet: Whether a person has access to the internet (e.g. without having to ask permission) |
| 6 | Acculturation | Acculturation: A process in which members of one cultural group adopt the beliefs and behaviors of another group |
| 7 | Active transport policy | Active transport policy: Whether or not an institution has an active transport policy |
| 8 | Activities organised by members of own community | Activities organised by members of own community: Whether members of the own community regularly organise activities like a feast, play streets,… |
| 9 | Advertising | Advertising: The activity or profession of producing advertisements for commercial products or services regarding sedentary behaviour |
| 10 | Aesthetics of design | Aesthetics of design: Aesthetics of the indoor design, how everything indoor looks like |
| 11 | Age | Age: The time that has passed since the day a person was born |
| 12 | Age at time of migration | Age at time of migration: The age of a person at the moment that person migrated from his country of birth |
| 13 | Ageism | Ageism: Stereotyping and discriminating against individuals or groups on the basis of their age |
| 14 | Awareness of consequences parents/teachers/children/familly/personal | Awareness of consequences of parents/children/family/personal: Whether a person is aware of the consequences of their own behaviour on their personal life/the life of their parents/the life of their teachers/the life of their children/… |
| 15 | Awareness of time spent sitting | Awareness of time spent sitting: Whether a person is aware of the time they spent sitting on a regular day |
| 16 | Behaviour of peers and friends | Behaviour of peers and friends: Whether the behaviour of peers and friends influences the behaviour of the person itself |
| 17 | Body composition (weight, muscle, fat) | Body composition: The components of which a body is composed, such as weight, muscle, fat |
| 18 | Body image | Body image: The subjective picture or mental image of one's own body |
| 19 | Building design | Building design: Whether the design of the building has an effect on the behaviour; for example: availability of elevators, stairs,… specifically for the workplace, schools,… (children/adults) |
| 20 | Burden of disease | Burden of disease: Problems that are associated with the disease which creates a heavy burden on the person itself |
| 21 | Busy lifestyle of carer | Busy lifestyle of carer: Whether or not the carer (for children/older adults) has a busy lifestyle (such as a lot of hobbies, almost no free time,…) |
| 22 | Car ownerschip | Car ownership: Whether a person (adult/older adult) owns a car or not |
| 23 | Car parking | Car parking: Whether parking space for a car is close by or far away (adult/older adult) |
| 24 | Carer occupation | Carer occupation: The job or profession of the carer (for children/older adults) |
| 25 | Carer status (age, education, employment, training ) | Carer status: The age, education, employment, training of the carer (for children/older adults) |
| 26 | Carers TV viewing time | Carers TV viewing time: The time that a carer watches TV on a regular day (for children/older adults) |
| 27 | Carers weight status | Carers weight status: Whether the carer (e.g. parents, grandparents, legal guardians, family members) has underweight, normal weight, overweight or obesity (for children/older adults) |
| 28 | Collectivist norms/attitudes | Collectivist norms/attitudes: When a group of people thinks a like about several themes |
| 29 | Commitments to and within family/expectations | Commitments to and within family/expectations: The dedication of a person towards his family |
| 30 | Communication of healthcare practitioners | Communication of healthcare practitioners: Whether or not healthcare practitioners are able to regularly communicate with the people from their community |
| 31 | Commuting distance of the carer | Commuting distance of the carer: The distance that the carer (e.g., parents, legal guardians, member of the family) needs to cross to get to work |
| 32 | Computer use in school/place of work | Computer use in school/place of work: Whether a person needs to use the computer often at school/workplace |
| 33 | Consumer Price Index | Economical diagnostic reflecting the increase in price of every goods |
| 34 | Convenience of public transport | Convenience of public transport: Whether or not it is easy for a person to use public transport |
| 35 | Cultural background | Cultural background: A reference to any culture or subculture that an individual identifies as his or her heritage or background |
| 36 | Cultural health beliefs about PA | Cultural health beliefs about PA: When one's health beliefs about PA is influenced by one's culture |
| 37 | Cultural norms | Cultural norms: The rules that a specific group uses for stating what is seen as appropriate and inappropriate behaviors, values, beliefs, and attitudes |
| 38 | Cultural requirements | Customs specific to a culture or and ethnic group |
| 39 | Cultural view of leisure time | Cultural view of leisure time: A person's free time depends on the specific culture one is in |
| 40 | Day of the week | Day of the week: Whether it is a weekday or a weekend day |
| 41 | Depending on others | Depending on others: The way in which a person depends on someone else to take care of him/her |
| 42 | Depression | Depression: Severe despondency and dejection, typically felt over a period of time and accompanied by feelings of hopelessness and inadequacy |
| 43 | Design of public spaces | Design of public spaces: Whether or not some features are available in the public spaces, such as trees, benches, walking paths,… |
| 44 | Differences in preferred mode of transportation (e.g. Irrelevancy of cycling for many ethnic women ) | Differences in preferred mode of transportation: Someone might choose to use a bicycle while someone else might choose to use the car |
| 45 | Disability | Disability: A physical or mental condition that limits a person's movements, senses, or activities |
| 46 | Eating disorders | Eating disorders: Problems with eating food such as anorexia or bulimia |
| 47 | Eating in front of TV | Eating in front of TV: Whether a person eats the main meals or snacks in front of TV |
| 48 | Economic stability | Economic stability: Economic stability refers to an absence of excessive fluctuations in the macroeconomy. An economy with fairly constant output growth and low and stable inflation would be considered economically stable |
| 49 | Economy | Economy: An economy or economic system consists of the production, distribution or trade, and consumption of limited goods and services by different agents in a given geographical location |
| 50 | Educational level | Educational level: The highest level of education that a person has received; e.g. primary school, high school, college, university |
| 51 | Employment status | Employment status: Whether or not a person has a job, or is unemployed/sick |
| 52 | Energy level | Energy level: The amount of energy a person has to engage in different activities (to work, to do the household,…) |
| 53 | Energy price | Energy price: The price one has to pay to buy electricity,… |
| 54 | Ethnicity | Ethnicity: The fact or state of belonging to a social group that has a common national or cultural tradition |
| 55 | Familly habits, modelling parents siblings | Family habits, modelling parents, siblings: The habits of the family, siblings regarding sedentary behaviour |
| 56 | Familly structure | Family structure: The combination of relatives that comprise a family |
| 57 | Family cohesion | Family cohesion: The emotional bonding that family members have toward one another |
| 58 | Family size | Family size: The amount of people a family consists of |
| 59 | Fashion | Trend in what is fashionable in society |
| 60 | Ffear of racism | Fear of racism: Whether a person is afraid to be confronted with racism because of the color of their skin |
| 61 | Flexibility offo working hours | Flexibility of working hours: Whether or not it is possible to start work and end work whenever it suits you best |
| 62 | Gas price | Gas price: The price one has to pay to buy gas |
| 63 | Gender divide | Gender divide: The differences between women and men, especially as reflected in social, political, intellectual, cultural, or economic attainments or attitudes |
| 64 | Generational divide | Generational divide: The difference of opinions on music, values, politics, et cetera, that occurs between one generation and another, usually between younger people and their parents and/or grandparents |
| 65 | Generational equality | Generational equality: The concept or idea of fairness or justice in relationships between children, youth, adults and seniors, particularly in terms of treatment and interactions |
| 66 | Genetic predisposition | Genetic predisposition: An inherited genetic pattern that makes one susceptible to a certain disease |
| 67 | Geographical isolation | Geographical isolation: Whether someone lives in a geographical area which is not easy to reach |
| 68 | Geriatric syndromes (pain, incontinence, fatigue, fear of falling, loss of strength ) | Geriatric syndromes: Syndromes that are associated with having an older age, such as pain, incontinence, fatigue, fear of falling, loss of strength |
| 69 | Green/blue areas | Proximity of area with greenery and water feature |
| 70 | Having a partner | Having a partner: A person (adult/older adult) who has a romantic relationship with another person |
| 71 | Having (grand) children | Having (grand)children: whether or not an individual has grandchildren |
| 72 | Health insurance, cost | Health insurance: whether a person has health insurance or whether a person can afford to have health insurance |
| 73 | Health status | Health status: the presence or absence of any disease or measures of functioning, physical illness, and mental wellbeing. |
| 74 | Identity | Identity: the fact of being who or what a person or thing is |
| 75 | Immigration history | Immigration history: whether or not a person is an immigrant or immigrated at some stage in their lives |
| 76 | Impairment | Impairment: the state of being diminished, weakened, or damaged, especially mentally or physically |
| 77 | Income | income: money received, especially on a regular basis, for work or through investments |
| 78 | Increased cost of movement (moving is energetically more demanding)? | Increased cost of movement: movement that takes more effort than is the norm - this could be related to numerous reasons e.g. physical impairment |
| 79 | Inflation | Inflation: a general increase in prices and fall in the purchasing value of money |
| 80 | Influence of peers and friends | Influence of peers and friends: the influence peers and friends have on an individual's behaviour |
| 81 | Institutional policy | Institutional policy: a course or principle of action adopted or proposed by an organisation |
| 82 | Interest rate | Interest rate: the amount charged, expressed as a percentage of principal, by a lender to a borrower for the use of assets |
| 83 | Job autonomy | Job autonomy: the degree or level of freedom and discretion allowed to an employee over his or her job |
| 84 | Karma/fatalism | Karma / fatalism: the belief that all events are predetermined and therefore inevitable |
| 85 | Language | Language: the system of communication used by a particular country or community |
| 86 | Leg power | Leg power: the strength and muscle control in one's lower limbs |
| 87 | Life experience of PA | Life experience of PA: the individual's life experience of physical activity |
| 88 | Life satisfaction | Life satisfaction: the perception of being happy with one's own life and a belief that one's life is on the right track |
| 89 | Living space design (home, dwelling) | Living space design: |
| 90 | Living with carer (parents included) | Living with a carer: living with a family member or paid helper who regularly looks after a child or a sick, elderly, or disabled person |
| 91 | Local authorities | Local authorities: an administrative body in local government that provides an extensive range of public services in an area |
| 92 | Locus of control | Locus of control: the extent to which an individual believes they can control events thsat affect them |
| 93 | Loneliness | Loneliness: a feeling of sadness due to the lack of friends or company |
| 94 | Media coverage portrayal of SB | Media coverage: the way in which a particular piece of information is presented by media either in a positive or negative light |
| 95 | Medication | Medication: a drug or other form of medicine that is used to treat or prevent disease |
| 96 | Mental health | Mental Health: an individual's condition with regard to their psychological and emotional well-being |
| 97 | Mobility issues | Mobility issues: issues relating to the ability to move or be moved freely and easily |
| 98 | Modelling the behaviour of others | Modelling behaviour: an individual learns new skills by imitating another person |
| 99 | Mortgage cost | Mortgage cost: the cost of an individual's mortgage |
| 100 | National Government | National government: the group of politicians or political parties that make up the government that run the country as a whole (as opposed to local government) |
| 101 | Necessity to use computer after work/school e.g. E-homework | Necessity to use PC: essential PC use |
| 102 | Neighbourhood nuissance (noise, pollution, degradation etc..) | Neighbourhood nuisance: an interference with neighbourhood enjoyment, either by creating smells, sounds, pollution or any other hazard that extends past the boundaries of one's property |
| 103 | Neighbourhood safety | Neighbourhood safety: the freedom in a neighbourhood from the occurrence or risk of injury, danger, or loss |
| 104 | Nneighbourhood walkability/cycle-ability | Neighbourhood walkability / cycle-ability: the measure of how friendly an area is to walking or cycling |
| 105 | Number of breaks during day occupation (school/work) | Numbers of breaks during the day: the number of breaks an individual's take during their normal day e.g. in school, in work |
| 106 | Number of kids in the house | Number of kids in the house: number of children living in the house |
| 107 | Number of screens in dwelling place | Number of screens in dwelling place: number of screens in the home or place of dwelling |
| 108 | Occupation | Occupation: an individual's job or profession |
| 109 | Outdoor playtime | Outdoor playtime: the amount of time spent outdoor playing |
| 110 | Over protecting peers/carers | Over protecting peers/ carers: peers or carers that are over protective |
| 111 | PA Guidelines | PA Guidelines: recommendations relating to physical activity levels |
| 112 | PA level (past & present) | PA levels: previous and current physical activity levels |
| 113 | Pain | Pain: a feeling of discomfort that interferes with everyday activities |
| 114 | Peers (friends or colleagues) | Peers: friends and colleagues |
| 115 | Pension | Pension: a regular payment made by the state to people of or above the official retirement age and to some widows and disabled people |
| 116 | Perceived health on the day | Perceived health on the day: the way one is feeling on a particular day |
| 117 | Perceived safety from crime | Perceived safety from crime: perceived control of recognised hazards in one's locality |
| 118 | Personality | Personality: the combination of characteristics or qualities that form an individual's distinctive character |
| 119 | Pet ownership | Pet ownership: owning a pet such as a cat or dog |
| 120 | Physical capacity/function | Physical capacity / function: an individual's ability to perform physically or functionally |
| 121 | Physical education | Physical education: instruction in physical exercise and games, especially in school |
| 122 | Physical fitness/skills | Physical fitness: a general state of health and well-being and, more specifically, the ability to perform aspects of sports |
| 123 | Physical organisation and furniture of place of education/work/care | Physical organisation and furniture of place of education / work / care: the physical and aesthetic design of place of education/work/care |
| 124 | Political stability | Political stability: a stable political system that survives through crises without internal warfare |
| 125 | Popular culture | Popular culture: ideas, customs and social behaviours that are popular |
| 126 | Positive social norm to screen use | Positive social norm to screen use: that it is socially acceptable to use screens for entertainment / work / study |
| 127 | Pregnancy | Pregnancy: the condition of carrying an embryo or feotus |
| 128 | Pressure (Academic/productivity) | Pressure: the use of persuasion or intimidation to make someone do something they may or may not want to do |
| 129 | Productivity | Productivity: the results of an individual’s effort measured as output |
| 130 | Provision of resting place | Provision of resting place: that resting places are provided |
| 131 | Psychology (attitude, temperament, motivation) | Psychology: the state of one's psychology including attitude, temperament and motivation |
| 132 | Public health Campaign | Public health campaign: an effort to persuade a defined public to engage in behaviors that will improve health or refrain from behaviors that are unhealthy |
| 133 | Public open spaces | Public open spaces: any open piece of land that is undeveloped (has no buildings or other built structures) and is accessible to the public, including playgrounds and seating areas |
| 134 | Public transport | Public transport: any transport including buses and trains, that are available to the public, charge set fares, and run on fixed routes |
| 135 | Quality of life | Quality of life: an individual's experienced standard of health, comfort, and happiness |
| 136 | Recreational facilities | Recreational facilities: any building or place that provides a particular service or is used for a particular industry e.g. football pitch or swimming pool |
| 137 | Religious belief and practice | Religious belief and practice: an individual's belief in a god or in a group of gods and practising life in accordance with such beliefs |
| 138 | Religiously required ways of dressing | Religiously required ways of dressing: dressing in accordance with religious teaching and beliefs |
| 139 | Retirement | Retirement: the action or fact of leaving one's job and ceasing to work |
| 140 | Risk adverse society | Risk adverse society: a society opposed to taking risks or only willing to take small risks |
| 141 | Risk behaviours (alcohol, tobacco, drugs) | Risk behaviours: lifestyle behaviours that potentially expose people to harm, or significant risk of harm which will prevent them reaching their potential |
| 142 | Role/behaviour modelling | Mimicing behaviour of others |
| 143 | Room (fresh air, temperature, light, music, colours, furniture, comfortability, number of persons) | General feel of a room where a person live/work |
| 144 | Room temperature | Ambient temperature of the living/working environment |
| 145 | Rules regarding TV time/computer | Rules enforced at home or in any living environment (e.g. care settings) about how much and when TV is watched |
| 146 | Rural living | Living in the countryside |
| 147 | Safe surroundings | Is the physical environment of a person physically and mentally safe for them |
| 148 | Sarcopenia | Mineral bone loss |
| 149 | SB level (past & present ) | What was the sedentary behaviour of a person in the past |
| 150 | School attendance | Is a child attending school |
| 151 | School satisfaction | How satisfied is a child or their parents with their school |
| 152 | Season | Time of the year (winter,spring,summer,automn) |
| 153 | Seated exercise | Exercise classes delivered in seated position |
| 154 | Seated hobbies | Person past time or interest which involve activities performed in sitting |
| 155 | Self-efficacy | [the extent or strength of one's belief in one's own ability to complete tasks and reach goals](http://en.wikipedia.org/wiki/Goal) |
| 156 | Self-esteem | [a person's overall subjective emotional evaluation of his or her own worth.](http://en.wikipedia.org/wiki/Person) |
| 157 | Self-rated health | How a person rate their own health |
| 158 | Sense of coherence | The extent to which one has a pervasive, enduring though dynamic, feeling of confidence that one’s environment is predictable and that things will work out as well as can reasonably be expected |
| 159 | SES | Social Economical Class |
| 160 | Shift pattern at work (eg night shift) |  |
| 161 | Shower at work | Whether showering facilities are available in the work place |
| 162 | Sleeping time | How long a person spends sleeping |
| 163 | Snacking | Eating food outside of meal hours |
| 164 | Social capital | Collective benefit from cooperative and preferential treatment between individual and groups |
| 165 | Social class | Cultural social class different from Social Economical class |
| 166 | Social desirability | Tendency to adopt societal trends norms |
| 167 | Social isolation | Lack of social connection |
| 168 | Social media | Computer mediated tools that allow people to network |
| 169 | Social network | The extend of social connection a person has. |
| 170 | Social Norms | Norms defining appropriate behavior within a group |
| 171 | Social role (working, caring, volunteering) | Whether a person assumes an active role within society through working, caring or volunteering |
| 172 | Social ties with home country (e.g. Watching homeland TV programmes) | For migrants or descendent of migrants pattern of behaviour such as watching homeland TV programmes |
| 173 | Societal financial resources | Financial wealth available to the state |
| 174 | Society/community social patterns | Trends in collective behaviour within a group of people (community or a whole society) |
| 175 | Street ligthts | Presence of lighting in the streets |
| 176 | Time with parents | Amount of time children spend with their parents (this irrespective of age) |
| 177 | Topography | Geophysical shape of the environment, hills, plains |
| 178 | Traffic safety | If road are safe to walk |
| 179 | Training of care providers | Whether or not a care provider (whether guardian or health professional) is trained adequately about sedentary behaviour or about physical activity |
| 180 | Transport infrastructures | Framework that support the transport system. This includes roads, railways, waterways, airports |
| 181 | TV in bedroom | Whether or not an individual has a TV in their bedroom |
| 182 | Type of housing | Whether it is communal, or single occupancy, house, flat, high rise |
| 183 | Type of TV programmes | Whether specific type of tv program are important to the individual (in migrants for example this might be an important cultural link) |
| 184 | Urban planning | Technical and political process concerned with the use of land and design of the urban environment |
| 185 | Values | Set of values associated with sitting. |
| 186 | Vision (eye) | Quality of eye vision, aquity, impairments |
| 187 | Weather |  |
| 188 | Women discouraged from leaving home unattained | In some ethnic, migrant, social group women might have to fulfil the role of house guardian |
| 189 | Work ethics | Value attached with hard work and diligence |
| 190 | Working culture and norms | norms and culture defining appropriate behaviour within the working environment |

Table S2: Factors identified in each cluster as having combined highest modifiability and population level effect size in youths

| Health and Well being | Social and Cultural settings | Behaviour and Psychology | Built and Natural Environment | Institutional/Home settings | Politics and Economy |
| --- | --- | --- | --- | --- | --- |
| \| - Physical fitness/skills \| \| --- \| \| - Energy level \| \| - Mental health \| | \| - Type of TV programmes \| \| --- \| \| - Media coverage portrayal of SB \| \| - Familly habits/modelling parents siblings \| \| - Social desirability \| \| - Time with parents \| \| - Pressure (Academic/productivity) \| \| - Carers TV viewing time \| \| - Positive social norm to screen use \| \| - Peers (friends or colleagues) \| \| - Behaviour of peers and friends \| \| - Social network \| \| - Cultural view of leisure time \| \| - Over protecting peers/carers \| \| - Cultural health beliefs about PA \| \| - Number of kids in the house \| | \| - Eating in front of TV \| \| --- \| \| - SB level (past & present ) \| \| - PA level (past & present) \| \| - Snacking \| \| - School attendance \| \| - Self-efficacy \| \| - Differences in preferred mode of transportation \| \| - Modelling the behaviour of others \| | \| - Safe surroundings \| \| --- \| \| - Recreational facilities \| \| - Design of public spaces \| \| - Neighbourhood safety \| \| - Urban planning \| \| - Neighbourhood walkability/cycle-ability \| \| - Green/blue areas \| | \| - Rules regarding TV time/computer \| \| --- \| \| - Computer use in school/place of work \| \| - Physical organisation and furniture of place of education \| \| - Outdoor playtime \| \| - Accessibility to the internet \| \| - Access to screen based devices \| \| - Pet ownership \| \| - Number of screens in dwelling place \| \| - TV in bedroom \| \| - Number of breaks during day occupation (school/work) \| \| - Access to garden \| \| - Institutional policy \| \| - Necessity to use computer after work/school e.g. E-homework \| \| - Type of housing \| \| - Commitments to and within family/expectations  - Social media \| | \| - Advertising \| \| --- \| \| - Physical education \| \| - Collectivist norms/attitudes \| \| - Public health Campaign \| |

**Table S3: Factors identified in each cluster as having combined highest modifiability and population level effect size in adults**

| Health and Well being | Social and Cultural settings | Behaviour and Psychology | Built and Natural Environment | Institutional/Home settings | Politics and Economy |
| --- | --- | --- | --- | --- | --- |
| \| - Physical fitness/skills \| \| --- \| \| - Increased cost of movement \| \| - Energy level \| \| - Loneliness \| \| - Sleeping time \| \| - Communication of healthcare practitioners \| | \| - Type of TV programmes \| \| --- \| \| - Social desirability \| \| - Eating in front of TV \| \| - Media coverage portrayal of SB \| \| - Familly habits/modelling parents siblings \| \| - Pressure (Academic/productivity) \| \| - Social Norms \| \| - Social network \| \| - Differences in preferred mode of transportation \| \| - Cultural health beliefs about PA \| \| - Commitments to and within family/expectations \| \| - Society/community social patterns \| \| - Body image \| | \| - SB level (past & present ) \| \| --- \| \| - Self-efficacy \| \| - Role/behaviour modelling \| \| - Modelling the behaviour of others \| \| - Personality \| | \| - Recreational facilities \| \| --- \| \| - Safe surroundings \| \| - Design of public spaces \| \| - Urban planning \| \| - Green/blue areas \| \| - Building design \| \| - Room (fresh air/temperature/light/music/colours/ furniture/comfortability/number of persons) \| \| - Public transport \| | \| - Computer use in school/place of work \| \| --- \| \| - Physical organisation and furniture of place of education/work/care \| \| - Rules regarding TV time/computer \| \| - Access to screen based devices (computer/computer/video games etc) \| \| - Accessibility to the internet \| \| - Pet ownership \| \| - Number of screens in dwelling place \| \| - TV in bedroom \| \| - Family cohesion \| \| - Institutional policy \| \| - Necessity to use computer after work/school e.g. E-homework \| \| - Type of housing \| \| - Geographical isolation \| \| - Productivity  - Social media \| | \| - Advertising \| \| --- \| \| - Active transport policy \| \| - Local authorities \| |

**Table S4: Factors identified in each cluster as having combined highest modifiability and population level effect size in older adults**

| Health and Well being | Social and Cultural settings | Behaviour and Psychology | Built and Natural Environment | Institutional/Home settings | Politics and Economy |
| --- | --- | --- | --- | --- | --- |
| \| **-** Ability to use health care \| \| --- \| \| **-** Availability of health care provision \| \| **-** Impairment \| \| **-** Leg power \| \| **-** Medication \| \| **-** Mobility issues \| \| **-** Physical capacity/function \| \| **-** Physical fitness/skills \| \| **-** Quality of life \| \| **-** Communication of healthcare practitioners \| \| **-** Depression \| \| **-** Energy level \| \| **-** Sarcopenia \| | \| **-** Activities organised by members of own community \| \| --- \| \| **-** Behaviour of peers and friends \| \| **-** Having a partner \| \| **-** Loneliness \| \| **-** Over protecting peers/carers \| \| **-** Peers (friends or colleagues) \| \| **-** Pet ownership \| \| **-** Carer occupation \| \| **-** Carers TV viewing time \| \| **-** Collectivist norms/attitudes \| \| **-** Commitments to and within family/expectations \| \| **-** Cultural health beliefs about PA \| \| **-** Cultural view of leisure time \| \| **-** Social class \| \| **-** Type of TV programmes \| | \| **-** Life satisfaction \| \| --- \| \| **-** Locus of control \| \| **-** PA level (past & present) \| \| **-** Perceived health on the day \| \| **-** Psychology (attitude/temperament/motivation) \| \| **-** Self-efficacy \| | \| **-** Green/blue areas \| \| --- \| \| **-** Neighbourhood safety \| \| **-** Neighbourhood walkability/cycle-ability \| \| **-** Perceived safety from crime \| \| **-** Recreational facilities \| \| **-** Design of public spaces \| \| **-** Safe surroundings \| \| **-** Transport infrastructures \| | \| **-** Access to garden \| \| --- \| \| **-** Access to screen based devices (computer/computer/video games etc) \| \| **-** Institutional policy \| \| **-** Living space design (home/dwelling) \| \| **-** Number of screens in dwelling place \| \| **-** Physical organisation and furniture of place of education/work/care \| \| **-** Eating in front of TV \| \| **-** Room (fresh air/temperature/light/music/colours/ furniture/comfortability/number of persons) \| \| **-** Seated exercise \| \| **-** Type of housing \| \| **-** TV in bedroom \| | \| **-** Advertising \| \| --- \| \| **-** Health insurance/cost \| \| **-** Local authorities \| \| **-** Public health Campaign \| \| **-** Car ownership \| |
